# Effects of Zinc supplementation on serum copper to Zinc and CRP to albumin ratios in hemodialysis patients

**DOI:** 10.5937/jomb0-26698

**Published:** 2021-03-12

**Authors:** Marwa Hajji, Rania Khedher, Mehdi Mrad, Hammami Mohamed Bassem, Nawel Rafrafi, Salma Chouchi, Moncef Feki, Afef Bahlous, Karim Zouaghi, Hayet Fellah

**Affiliations:** 1 University of Tunis El Manar, Faculty of Medicine of Tunis, Tunis, Tunisia; 2 Rabta University Hospital, Laboratory of Biochemistry, Tunis, Tunisia; 3 Rabta University Hospital, Department of Nephrology, Tunis, Tunisia; 4 Pasteur Institute, Laboratory of Biochemistry, Tunis, Tunisia; 5 Radial Hemodialysis Clinic, Manouba, Tunisia; 6 Udial Hemodialysis Clinic, Tunis, Tunisia

**Keywords:** copper to zinc ratio, C-reactive protein to albumin ratio, hemodialysis, zinc supplement, odnos bakra prema cinku, odnos C-reaktivnog proteina i albumina, hemodijaliza, suplement cinka

## Abstract

**Background:**

Zinc (Zn) deficiency is a common condition and could contribute to poor outcomes in hemodialysis (HD) patients. The aim of this study was to evaluate the effects of Zn supplementation on serum copper (Cu) to Zn and C-reactive protein (CRP) to albumin ratios (CAR) in HD patients.

**Methods:**

Seventy-seven HD patients were enrolled in a multicentre simple-blind randomized clinical trial. Only 37 HD patients completed the study; they were randomly divided into two groups and supplemented with zinc sulphate (n=17) or placebo (n=20) for two months. Serum Zn and Cu were measured by atomic absorption spectrophotometry. Serum albumin and hypersensitive-CRP were assessed by colorimetric and immunoturbidimetric method, respectively. Determinations were performed before and after supplementation.

**Results:**

After two months of supplementation, serum Zn significantly increased, and Cu to Zn ratio decreased in Zn supplemented group, but remained unchanged in the placebo group. In parallel, serum albumin concentrations significantly increased, and CAR decreased in Zn supplemented group only.

**Conclusions:**

Zn supplementation reduces Cu to Zn and CRP to albumin ratios in HD patients. These changes point towards an improvement in nutritional, oxidative and inflammatory status. The study findings suggest that correcting Zn deficiency reduces poor outcomes in HD patients.

## Abstract (sr)

**Uvod:**

Manjak cinka (Zn) je uobičajeno stanje i može doprineti lošim ishodima kod pacijenata na hemodijalizi (HD). Cilj ove studije je bio da proceni efekte suplementacije Zn na odnos serumskog bakra (Cu) i Zn i odnos C-reaktivnog proteina (CRP) i albumina (CAR) kod pacijenata na hemodijalizi.

**Metode:**

Sedamdeset i sedam pacijenata na hemodijalizi je uključeno u jednostruko - slepo i randomizovano kliničko ispitivanje u više centara. Samo 37 pacijenata na hemodijalizi je završilo studiju. U nasumično podeljene dve grupe počelo se sa suplementacijom u ishrani cink sulfatom (n=17) ili placebom (n=20) tokom dva meseca. Serum Zn i Cu su mereni atomskom apsorpcionom spektrofotometrijom. Albuminski serum i hipersenzitivni CRP procenjeni su kolorimetrijskim i imunoturbidimetrijskim metodom, svaki posebno. Merenja su vršena i pre i posle suplementacije.

**Rezultati:**

Nakon dva meseca suplementacije Zn u serumu se značajno povećao, a odnos Cu i Zn smanjio u grupi gde je sprovođena suplemenacija Zn, ali je ostao nepromenjen u grupi sa placebom. Paralelno sa tim, koncentracije albumina u serumu značajno su porasle, a CAR se smanjio samo u grupi sa suplementacijom Zn.

**Zaključak:**

Suplementacija Zn smanjuje odnos Cu u odnosu na Zn, i CRP u odnosu na albumin kod pacijenata na hemodijalizi. Ove promene ukazuju na poboljšanje statusa u vezi sa ishranom, oksidacijom i zapaljenjima. Nalazi studije sugerišu da smanjenje deficita Zn umanjuje šanse za loše ishode kod pacijenata na hemodijalizi.

## Introduction

HD is associated with a high burden of poor cardiovascular outcomes and increased mortality [1]. This has been associated with conditions such as malnutrition, inflammation and oxidative stress [2] [3]. Zn is a trace element that is essential for human health. It regulates numerous enzyme activities and immune function and exerts antioxidant and anti-inflammatory effects [4] [5]. It is involved in proteins and lipids metabolism, nucleic acids synthesis and oxygen transportation [6] [7]. Zn deficiency, a common condition in HD patients, is associated with malnutrition, inflammation and oxidative stress [6] [7] [8] [9] and thus could be an additional contributor to poor outcomes in HD patients [10]. Cu, another trace element, has a role in haemoglobin synthesis, immune function and defence against oxidants [11]. It was shown that Cu and Zn interact antagonistically at high levels [12]. Serum Cu to Zn ratio is considered a better marker for inflammation, oxidative stress and cardiovascular risk than serum Zn or Cu alone [3] [13]. Elevated serum CRP and hypoalbuminemia are common in HD patients and were associated with adverse health effects in these patients [14] [15] [16]. CAR is a biomarker of inflammation and nutrition. Elevated CAR reflects increased inflammation superimposed with malnutrition. It was shown to predict poor outcomes and increased mortality risk in patients with severe sepsis [17], cancer [18], acute coronary syndrome [19] as well as patients undergoing HD [20].

Zn supplementation was proved to ameliorate nutritional, inflammatory and oxidant status [9] [21] [22]. A previous study showed a decrease in Cu to Zn ratio in patients under dialysis following Zn supplementation [9]. However, the impact on CAR has never been investigated. The present study aimed to examine the effect of Zn supplementation on serum Cu to Zn and CRP to albumin ratios.

## Material and Methods

A multicentre randomized single-blind clinical trial was conducted among 77 HD patients (45 men and 32 women) from February 2017 to March 2018. The patients were recruited from five centres: Rabta Hospital Nephrology department, Matri Hospital HD unit, Manouba HD Radial centre, Manouba HD public centre as well as HD Udial centre. An individual clinical card was established on which personal data were collected. The eligibility criteria for patients to be enrolled were over 18 years of age, HD treatment for at least six months, HD performed three times per week (each for 4 hours) through a polysulfone membrane against a dialysis liquid containing the following ions: Na^+^: 138 mmol/L; K^+^: 2 mmol/L; Ca^++^: 1.5 mmol/L; Mg^++^: 0.5 mmol/L; Cl^-^: 109 mmol/L; CH_3_CO_2_^-^: 3 mmol/L; HCO_3_^-^: 35 mmol/L. The clearance of urea evaluated by the ratio Kt/V was 1.2. The exclusion criteria were infection, gastrointestinal and liver diseases, congestive heart failure, cancer, psychiatric illness, pregnancy, use of immunosuppressants, corticoids, estrogens or contraceptives, as well as active smoking and alcoholism.

**Figure 1:**
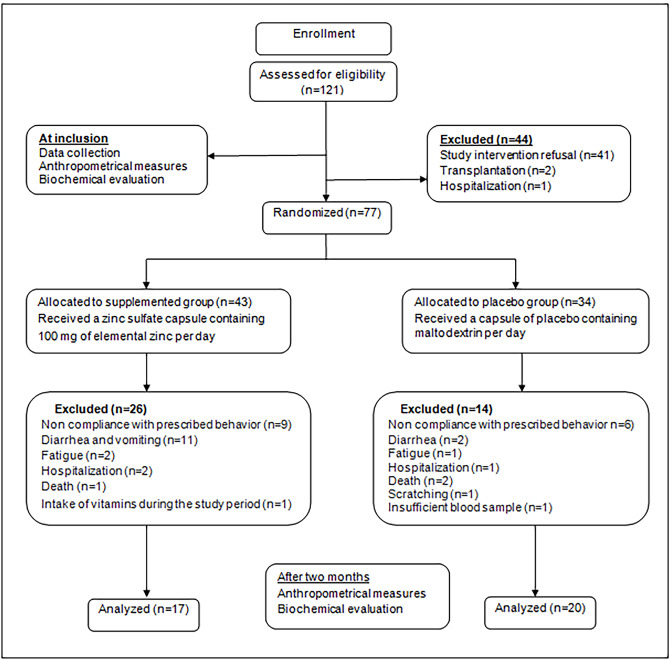
Flow chart for the process of hemodialysis patients selection.

The patients were randomly divided into two groups; 43 HD patients received one oral capsule containing 220 mg of Zn sulphate (100 mg of elemental Zn) (Zn group), and 34 HD patients received one oral capsule of similar appearance containing 220 mg of maltodextrin. Capsules were taken daily after dinner during 60 consecutive days. Randomization was performed while stratifying on gender, 5-year age class and duration of HD. Predialysis blood samples were obtained from all patients after a fast of 12 hours at inclusion (day 1) and after 60 days of supplementation (day 61). Serum Zn and Cu concentrations were determined by atomic absorption spectrophotometry (Perkin Elmer, Waltham, USA). Serum hypersensitive-CRP concentrations were determined by the immunoturbidimetric method using the Architect CI8200 auto analyser (Abbott Diagnostics, Chicago, USA). Serum albumin was assessed by colorimetric method using Cobas Integra 400 auto-analyser (Roche Diagnostics, Meylan, France). The study protocol was approved by the Ethics committee of Rabta Hospital and all patients gave their informed and signed consent to participate in the study. A total of 37 HD patients (17 in the supplemented group and 20 in the placebo group) completed the trial Figure 1.

### Statistical analysis

Statistical analyses were performed using the software package SPSS 22.0 for windows (SPSS Inc, Chicago, USA). Depending on the distribution of variables, data were reported as mean ± standard deviation (SD) or median (interquartile range). Between-group comparisons were made using independent t-test or Mann Whitney test, as appropriate. Within-group comparisons of variables before and after supplementation were performed using paired t-test or Wilcoxon rank test, as appropriate. A p-value <0.05 based on two-sided calculation was considered significant.

## Results

**Figure 2:**
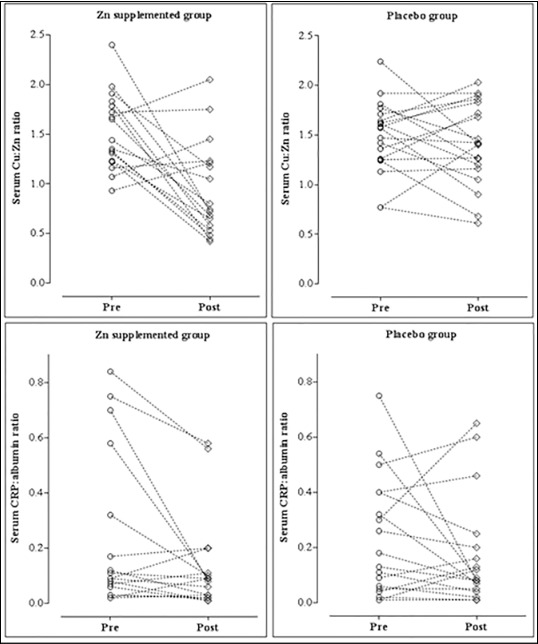
Changes in serum copper to zinc and C-reactive protein to albumin ratios in hemodialysis patients after two months of zinc supplementation.

Baseline clinical and biochemical characteristics, including serum Zn concentrations and prevalence of hypozincemia (serum Zn<800 μg/L), were comparable in Zn and placebo groups (Table 1). After two months of supplementation, serum Zn concentrations increased and serum Cu concentrations and Cu to Zn ratio decreased in the Zn group but remained unchanged in the placebo group. Zn supplementation also resulted in a significant increase in serum albumin and decrease in CAR. Figure 2 shows an individual variation in Cu to Zn and CRP to albumin ratios under supplementation in Zn and placebo groups.

**Table 1:** Baseline clinical and biochemical characteristics in zinc supplemented and placebo groups of hemodialysis patients. Values are expressed as mean ± SD or median (interquartile range); CRP, C-reactive protein; *, serum Zn <800 μg/L

	Zinc group (n=17)	Placebo group (n=20)	p value
Mean age, years	52.9±13.5	53.6±15.8	0.89
Male gender,%	70.6	55.0	0.33
Duration of	6.65±3.93	6.55±3.73	0.93
Body mass index, kg/m^2^	25.2±4.34	26.4±5.74	0.53
Serum creatinine, µmol/L	967±264	896±361	0.50
Serum albumin, g/L	37.1±2.17	37.2±3.62	0.90
Serum CRP, mg/L	3.90 (14.9)	5.90 (13.95)	0.85
Serum CRP to albumin ratio	0.09 (0.41)	0.15 (0.35)	0.88
Serum zinc, µg/L	768±166	812±168	0.42
Zinc deficiency *, %	64.7	55.0	0.55
Serum copper, µg/L	1160±332	1170±281	0.91
Serum copper to zinc ratio	1.52±0.38	1.47±0.35	0.62

## Discussion

Our study showed that Zn deficiency is common in Tunisian HD patients with a prevalence of 59.5%. This result is consistent with the findings of previous studies which reported Zn deficiency in more than 50% of HD patients [23] [24] [25]. Hypozincemia may result from Zn removal during HD treatment, reduced gastrointestinal absorption, low dietary intake and increased urinary excretion as well as protein restriction, increased expression of intracellular metallothioneins, and multi-infections [3] [21] [26].

Zn supplementation in these HD patients resulted in an increase in serum Zn and a decrease in Cu to Zn ratio, which is also consistent with literature data [9] [21]. If the improvement in Zn status following Zn supplementation is expected and comprehensible, the decrease in serum Cu could be explained by competition between the two trace elements at the phases of intestinal absorption and cellular trafficking. Indeed, Zn and Cu share the same enterocyte membranes' transporters and intracellular trafficking proteins [11] [12]. The competition may explain the decrease in Cu to Zn ratio following Zn supplementation. A high Cu to Zn ratio reflects oxidative stress, increased inflammation and immune dysfunction [12] [13] [27]. Thus, its reduction in HD patients is considered beneficial, which supports the usefulness of Zn supplementation in these patients.

The study also showed an increase in serum albumin and a reduction in CAR following Zn supplementation. Zn and Cu are known to influence the function of immune cells and the secretion of cytokines [26]. Zn exerts anti-inflammatory effects through downregulation of NF-B, leading to a reduction in proin ammatory cytokines [28]. Zn deficiency was shown to induce a decrease in CD4: CD8 ratio and the number of B-cells [29]. However, Zn supplementation was demonstrated to increase cells expressing CD19 [9]. Cu excess is considered potentially proinflammatory by inducing oxidative stress [30] [31]. CAR combines levels of CRP and albumin, a positive and a negative protein of acute phase inflammation, respectively. The reduction in the inflammatory response associated with Cu to Zn ratio decrease may inhibit the synthesis of positive proteins of acute phase inflammation including CRP and stimulate the synthesis of negative proteins of acute phase inflammation such as albumin. Our results are consistent with the findings of previous studies which reported a reduction in serum CRP concentrations [9] [21] and an increase in serum albumin after Zn supplementation [9] [32]. However, no previous study has investigated the impact on the ratio in HD patients.

The study has some limitations. First, the small sample size might have lowered the statistical power of the study. In fact, a number of patients in both Zn and placebo groups dropped out from the trial for lack of compliance with the study design or due to adverse health effects. HD patients were supplemented without taking into account their baseline serum Zn concentrations. This might explain some side effects that occurred in some patients. Further, trials with large sample size in Zn deficient patients are needed to confirm our results.

In conclusion, Zn supplementation in HD patients resulted in a decrease in Cu to Zn and CRP to albumin ratios. Such modifications point towards an improvement of nutritional, inflammatory and oxidative status. The study findings suggest that Zn supplementation is beneficial in HD patients and may contribute to reduce poor outcomes and improve the survival and quality of life in these patients. Therefore, patients on HD should be screened for Zn deficiency, and any deficiency should be adequately corrected.

## List of abbreviations

CAR, CRP to albumin ratio; CRP, C-reactive protein; HD, hemodialysis.

## Acknowledgements

The authors gratefully acknowledge the patients and administrative, nursing and technical staff for their valuable help. Special thanks to »Vital Laboratory« for providing Zn sulphate and placebo.

The study was supported by funds of Research Laboratory LR99ES11, Ministry of Higher Education and Scientific Research of Tunisia.

## Conflict of interest statement

The authors declare that they have no conflicts of interest in this work.
